# Lessons from using the Normalisation Process Theory to understand adherence to guidance on MgSO_4_ in preterm labour

**DOI:** 10.1186/s43058-025-00758-1

**Published:** 2025-07-16

**Authors:** Christalla Pithara-McKeown, Tracey Stone, Emma Treloar, Jenny Donovan, Karen Luyt, Sabi Redwood

**Affiliations:** 1 The National Institute for Health and Care Research Applied Research Collaboration West (NIHR ARC West) at University Hospitals Bristol and Weston NHS Foundation Trust, Bristol, UK; 2 Bristol Medical School, Population Health Sciences, University of Bristol, Bristol, UK; 3 University Hospitals Bristol and Weston NHS Foundation Trust, Bristol, UK

**Keywords:** Quality improvement, Normalisation process theory, Knowledge mobilisation, Magnesium sulphate, Preterm labour, Evidence-based guidelines

## Abstract

**Background:**

The administration of magnesium sulphate (MgSO_4_) in preterm labour is an evidence-based intervention recommended by the United Kingdom’s National Institute for Health and Care Excellence (NICE) to prevent neurological damage to the infant. However, uptake varies across UK maternity units. We used findings from three studies in England, Scotland and Wales investigating implementation of guidance on MgSO_4_ as neuroprotectant in preterm-labour to understand how knowledge mobilisation can drive scaling and spread of improvement.

**Methods:**

Remote semi-structured interviews were carried out as part of an evaluation of (1) the PReCePT (Preventing Cerebral Palsy in Pre-Term Labour) National Programme, and (2) the PReCePT cRCT study, and as part of a qualitative study investigating MgSO_4_ guidance implementation in Scotland and Wales. Normalisation Process Theory informed data collection and analysis. Data were analysed using the framework method.

**Results:**

Interviews with 86 strategic and clinical leads and implementers from the three nations suggested that despite evidence being necessary and important for policy decision-making and clinical buy-in, improvement interventions were motivated by audit data and benchmarking. Scaling of improvement was driven by knowledge sharing, diffusion of innovation, and capacity building through relational structures (e.g. networks, communities) spanning the perinatal ecosystem. Local champions operating in multiple communities and networks as boundary-spanners connected national and regional leadership, patient group representatives, implementers i.e. clinical leads and champions, and perinatal clinical teams to enable knowledge mobilisation. Their work relied on backfill funding and protected time, and social-cognitive and social-structural resources in their settings. Sense-making, cognitive participation, collective action and reflexive monitoring work took place iteratively and dynamically within and across these structures on each level of the system.

**Conclusions:**

QI interventions driven by knowledge mobilisation can drive scaling and spreading of improvement, but require knowledge sharing and an infrastructure within the system to support improvement capacity building. Strong leadership with the ability to address power imbalances between co-actors, and secure protected funding for local champions is also required.

**Supplementary Information:**

The online version contains supplementary material available at 10.1186/s43058-025-00758-1.

Contribution to the literature • Some settings are better than others at embedding evidence-based guidance in routine practice. Understanding how best to scale and spread evidence-based interventions can address disparities in clinical practice.

• Using the Normalisation Process Theory, we show implementation work must engage actors from all levels of the perinatal ecosystem, and relies on knowledge-sharing and collaboration within diverse networks and CoPs

• Communities of practice linked to system-wide networks can be a platform for scaling improvement.

• Local champions with funded and protected time, boundary spanners to link actors across system levels, rich team capital, leadership oversight, and knowledge mobilisation capacity-building can address disparities between settings.

## Background

Improving maternity care is a global priority but progress has been slow [1]. Despite evidence of best practice, efforts to scale and spread good practice and innovation are not always successful [2–4]. There are disparities within and across health care settings in quality of care and in clinical outcomes, and these disparities are linked to social determinants of health [5–7]. Clinical teams’ capacity to implement and embed evidence-based interventions and innovations in routine practice is one factor contributing to these disparities, making context central to implementation success [6, 8].

In the West of England, to address low uptake of neonatal magnesium sulphate (MgSO_4_) a team of neonatal, obstetric, midwifery staff, mothers, and Quality Improvement (QI) experts co-designed and implemented a QI intervention, the PReCePT project (Prevention of Cerebral Palsy in PreTerm Labour) [9]. Following its success, NHS England funded a national scale up initiative (National PReCePT Programme or NPP) to implement PreCePT in all maternity units in England. An embedded cluster Randomised Controlled Trial (cRCT) investigated level of implementation support needed locally to maximise uptake of MgSO_4_ – PReCePT Study [10]. PReCePT was found to be effective and cost-effective in improving and sustaining performance over time irrespective of intensity of implementation support provided to units, demonstrating how a QI toolkit implemented by funded local champions supported by QI and clinical leads can drive uptake of evidence-based interventions across settings [11–14].

Even though no difference was found in MgSO_4_ uptake, a qualitative process evaluation showed perinatal clinical microsystem characteristics such as teamworking and shared governance, and organisational improvement capability [15] at the start of implementation shaped success [16]. Where teams and organisations demonstrated low teamworking culture and low relational resource, enhanced support helped in redistribution of roles, closer collaboration, and knowledge-sharing – i.e. relational restructuring [17] –which in turn drove collective action [13, 16]. Relational restructuring was placed at the centre of spread and scale up activities [13] as a prerequisite for embeddedness and sustainment of perinatal evidence-based interventions [16].

Knowledge mobilisation (KMb) refers to processes by which research-based knowledge is accessed, applied and embedded into routine practice and can be a bridge between evidence and practice [18]. It relies on collaboration and engagement of all interest holders (e.g. clinicians, researchers, patients, members of the public, and managers) in sharing knowledge and co-creation of learning; local improvement activities can emerge out of knowledge-sharing processes making QI a component of KMb [19].

Relational processes including KMb and collaboration-based approaches such as communities of practice (CoP) and networks are popular implementation strategies [19–21]. How CoP can drive KMb has been well-researched (e.g. [20]), but how KMb and collaboration-based approaches can drive system-wide scaling and spreading of improvement has not. We use findings from the PReCePT evaluations, and an exploratory qualitative study looking into MgSO_4_ guidance implementation in Scotland and Wales where PReCePT was not implemented, to understand scaling and spreading mechanisms focusing on KMb processes. We use the Normalisation Process Theory (NPT) because of its explanatory power when investigating implementation mechanisms [22], but at the same time its ability to zoom in on contextual enablers and challenges driving differences in local performance [17]. NPT is a sociological theory which identifies, characterises, and explains key mechanisms promoting and inhibiting implementation, embeddedness, and integration (normalisation) of complex interventions into routine practice [23].

We now provide an overview of the PReCePT Programme, PReCePT study, and more recent work in the two Devolved Nations, all of which investigated implementation of MgSO_4_ guidance.

### Implementation of MgSO_4_ guidance in England, Scotland and Wales

PReCePT QI intervention is a co-developed toolkit of products and implementation guidance to create implementation resource within settings and drive change e.g. staff competencies, documentation, processes and procedures. Locally, PReCePT was implemented by funded midwifery champions. Academic Health Science Networks (AHSNs) led national scale up of PReCePT and provided clinical and implementation leadership and support through Patient Safety Collaboratives. Patient Safety Collaboratives worked in partnership with Maternity and Neonatal Safety Improvement Programme (MatNeo SIP) National Patient Safety teams [24].

All units participating in the NPP were invited to the PReCePT study, and be randomised in either the intervention – i.e. receive an enhanced support package delivered by QI coaches – or control arm of the cRCT, which received the standard level of support delivered by AHSNs [10]. An overview of these two interventions is in Supplementary Materials.

PReCePT was effective and cost-effective in improving uptake irrespective of level of implementation support, [11, 13, 14]. Further research was carried out to compare performance in England with Scotland and Wales, where PReCePT was not implemented (Devolved Nations Study) [14]. Even though uptake appeared to improve faster in England after the NPP launch, both Scotland and Wales showed improvements over the same period, raising the need to further understand the mechanisms behind spread of improvement across the three nations. To address this question, a qualitative study explored implementation strategies and interventions employed in the Devolved Nations [14].

We use findings from these three strands of research to answer the following questions: What was the role of KMb in driving improvement in the three nations? How do relational structures such as CoP and clinical networks drive co-creation and diffusion of knowledge across the system? And what are the challenges and enablers of KMb?

## Methods

### Design and recruitment

A detailed description of the mixed-methods evaluation of the NPP and PReCePT Study, and Devolved Nations study methodology can be found elsewhere [10, 13, 20–22].

For the qualitative process evaluations of the NPP and PReCePT study we conducted semi-structured telephone interviews with:

1. AHSN QI managers who provided implementation leadership and delivered the standard support package to units within their catchment areas as part of the NPP.
2. Regional obstetric or neonatal clinical leads enrolled by AHSNs to provide clinical leadership to units.
3. Champion midwives and clinical lead obstetricians and neonatologists working in units randomised to the control arm of the cRCT, and received standard level of support from AHSNs.
4. Champion midwives and clinical lead obstetricians and neonatologists working in units randomised to the cRCT intervention arm and received enhanced level of support from the PReCePT Study management team and QI coaches.

The Devolved Nation study explored implementation practice in the devolved nations. We were guided by a steering group which included clinical leads from national perinatal clinical networks from the two nations to get a sense of implementation policies and interventions at a national clinical network level. We carried out semi-structured remote (MS Teams) interviews with:

1. Perinatal network leads involved in strategic planning of national clinical guidance implementation activities.
2. National leads responsible for implementing national QI initiatives.
3. Local clinical leads involved in improvement efforts and implementation of clinical guidance.

Recruitment was guided by the concept of information power [25] which suggests that number of participants recruited is determined by the richness of responses and the depth of participants’ knowledge on the topic of interest.

### Data collection and analysis

For the PReCePT study we devised two topic guides for enhanced and standard support units. We explored views of the PReCePT Toolkit, implementation support received, barriers and enablers to engaging in support activities and implementation, use of QI methods and using data for improvement. For the NPP we used the same topic guide for interviews with champions and local clinical leads, and a separate one for implementation strategic leads (AHSN managers and regional clinical leads). For the devolved nations one topic guide was used for all implementers with minor adjustments depending on their strategic or implementation leadership role to explore MgSO_4_ guidance implementation activities and factors impacting on implementation.

**Fig. 1:**
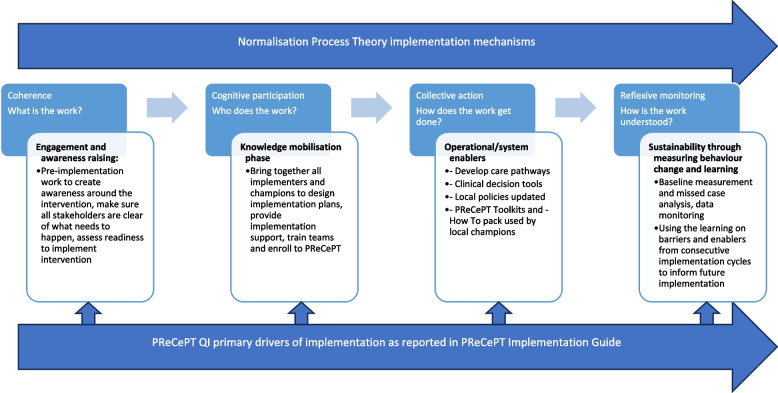
The Normalisation Process Theory implementation mechanisms and how they overlap with the four primary drivers of PReCePT

For PReCePT, we developed an analytical framework informed by research objectives, but as analysis progressed we identified PReCePT implementation drivers, i.e. the *function* of its components, mapped out onto NPT mechanisms [26] (Fig. 1). Contextual and intervention enablers and challenges reflected social-cognitive, and social-structural resources as captured by the Capacity and Potential components of the NPT [27]. We amended our analytical framework as per NPT coding manual for qualitative research [17]. This framework was used for analysing the Devolved Nations study data.

Data for the NPP and PReCePT Study were collected between July 2019 and December 2020 by CP-McK and TS using telephone interviews. The geographical distribution of participants across England made face-to-face interviews impractical. PReCePT data collection was pre-COVID19 so data collection using video platforms was not an option. Data for the Devolved Nations study were collected between November 2022 and July 2023 by CP-McK. Video and telephone interviews were given as options to participants, but the large majority chose video, unless connection was an issue.

Interviews lasted between 28 and 58 minutes, with the average interview lasting 33 minutes.

All interviews were audio-recorded with participants’ consent, transcribed, and analysed in QSR NVivo using the framework method [28]. This method provides a systematic model for managing and mapping data through a matrix capturing cases and themes, and allows for comparing and contrasting within and between cases [28]. Two researchers (CP-McK and TS for the PReCePT studies; CPMcK and KS for the Devolved Nations study) independently coded three transcripts and discussed discrepancies in the coding to inform development of a coding framework. Emerging findings were regularly presented to steering group members to discuss relevance, identify gaps, and inform subsequent data collection.

## Findings

For the NPP we interviewed 13 midwives, 11 obstetricians, 10 neonatologists, nine regional clinical leads, and 12 AHSN staff responsible for providing QI support to champions. For the PReCePT study we interviewed 9 midwife champions, 4 obstetric leads, and 5 neonatal leads.

For the Devolved Nations study, 8 participants from Wales, and five from Scotland were recruited. Six held national strategic leadership roles. Table 1 describes participant characteristics in more detail.

**Table 1:** Participant information

National PReCePT Programme	13 midwives 11 obstetricians 10 neonatologists 9 regional clinical leads 12 AHSN improvement managers Total = 55
PReCePT study (Intervention arm)	9 midwives 4 obstetricians 5 neonatologists Total = 18
Devolved Nations: Wales	4 neonatologists 1 Advanced Neonatal Nurse Practitioner 2 Obstetricians 1 Midwive Total = 8 4 held National strategic clinical leadership roles 1 held Champion role 2 held Improvement roles 2 held Clinical lead roles within their organisation
Devolved Nations: Scotland	2 Neonatologists 2 Obstetricians 1 Advanced Neonatal Nurse Practitioner *Total* = 5 1 held National strategic implementation (MCQIC) role 1 held National implementation (MCQIC) role 1 held National strategic clinical leadership role 2 held Regional implementation roles

### Implementation context

Neonatal MgSO_4_ has been embedded in National Institute for Health and Care Excellence (NICE) preterm labour and birth guidance since 2015, but its implementation is not a statutory requirement. Healthcare in the UK is a devolved responsibility, meaning each of the four nations – England, Northern Ireland, Scotland, and Wales – sets its own healthcare policies and delivers services to its population. Up until 2017 just before PReCePT was scaled nationally, uptake in England, Scotland and Wales was 64.1%, with regional variations ranging between 49%−78.8% [11]. Supplementary Information File 1 provides an overview of the national strategies and interventions employed by the three nations to implement MgSO_4_ into practice, as emerged from data analysis.

Across nations maternity and neonatal clinical networks merged and now function as perinatal collaborative partnerships, which alongside the British Association of Perinatal Medicine (BAPM) cultivated a perinatal teamworking culture and communication across geographical settings, organisations, disciplines and roles.

Input from members of the steering committee and data analysis highlighted how definitive evidence alongside embeddedness of the intervention in clinical guidance galvanised support from senior clinicians, but it was audit data and performance which mobilised clinical and strategic leads into action [13, 14, 16]. In England, statutory bodies and requirements such as the Clinical Negligence Scheme for Trusts, Maternity and Neonatal Safety Improvement Programme (MatNeo SIP), and operational and delivery remits of clinical networks provided strong incentives for organisations and teams to prioritise and commit to perinatal optimisation and improvement [16] but these were not mirrored in the two nations. Scotland implemented MCQIC PPWP in partnership with Scottish NHS boards in 2017. This was led by a clinical and QI team centrally, with coaches overseeing and supporting local implementers. Training was also provided. Implementation responsibility was decentralised at a later stage and local health boards then led on activities. This was around the same time that national and professional bodies were embedding MgSO_4_ evidence in their guidelines i.e. NICE 2015, followed by the Royal College of Paediatrics and Child Health, and Royal College of Obstetrics and Gynaecology guidance. PReCePT was designed and implemented in the West of England in 2014, and scaled up in 2018. PERIPrem (Perinatal Excellence to Reduce Injury in Premature Birth – a perinatal care bundle of 11 interventions) superseded PReCePT in 2020 [29] and implemented across the South West of England. In 2023 implementation efforts led by Wales’s Strategic Maternity and Neonatal Network culminated in the national roll out of PERIPrem, funded by the Welsh Government. At the time of data collection, Scottish MCQIC PPWP was being updated into the Scottish Patient Safety Programme (SPSP) Perinatal Programme.

### Coherence

Coherence refers to how individuals understand and make sense of a change to practice, such as administration of MgSO_4_ to women in preterm labour. This involves understanding how individuals think the change differs from current practices, how they assess its impact on their roles and responsibilities, and how they value it [17].

Despite evidence and clinical guidance on MgSO_4 _administration, these were operationalised and interpreted differently in obstetric and neonatal professional guidelines. Expertise and clinical governance responsibilities were held by neonatologists, and adopters i.e. maternity teams, and especially midwifery and junior staff, were not always involved on equal terms in training, education, and implementation activities. Power imbalances and lack of professional agency by members of the maternity team created challenges for administration and for securing implementation buy-in. Creating a shared sense of ownership, awareness of and consensus on clinical pathways and workflows among all team members was a priority among Devolved Nation clinical leads as illustrated by the following excerpt:

*A lot of it was understanding the why we want you to change your behaviours, and if you don’t know the evidence why would you change your behaviour? So, I think it’s having those shared common goals across all of our specialties, and building the team from that joined-up approach from the start, and not just working in a silo. (P13, Neonatologist, Scotland)*

Clinical and professional networks created relational infrastructure – e.g. communication and collaboration platforms – for knowledge sharing, which opened channels for innovation and good practice to diffuse across the system e.g. toolkits. PReCePT and PERIPrem, and BAPM perinatal optimisation toolkits informed implementation activities in Scotland and Wales, such as clinical guidance and pathways, to create coherence among maternity and neonatal teams. Implementation on the ground was made challenging, however, by the clinical nuances and complexities of preterm labour. Maternity staff lacked clarity on what the guidance or toolkit products meant in practice for them. Champions supporting frontline staff needed sense-making support from clinical leads to address clinical uncertainties, and QI capacity-building and mentoring to drive implementation in their settings. Opportunities and protected time for champions to attend cross-organisational CoP facilitated by clinical and QI leads before and after local implementation launch helped co-create meaning on what implementers were expected to do and how, especially specific to their local contexts, as the following excerpt illustrates:

*Once we’d done the meetings, I knew exactly what we were doing. We’d been and had our first meeting and I became aware of the QI Toolkit which had lots of resources on there that you could print off and use, you could adapt them to suit your Trust as well.[…] They were very informative. […] but (also helped with) sort of people managing (P51, Midwife, Standard Support)*

### Cognitive participation

Cognitive participation is the relational work people do to build and sustain a community of practice around a complex intervention, the space where knowledge sharing and co-creation take place. It also relates to people’s understanding of their role and that of their team in implementing the intervention [17].

Participants highlighted multi-professional teamworking as core for perinatal optimisation, but collaboration was hindered by staff shortages, organisation funding pressures, commissioning and funding mechanisms, and a culture of silo-working. Devolved Nation participants referred to external reports such as the Ockenden report, and a perinatal teamworking drive as galvanising organisations and teams to make collaboration and teamworking a strategic priority. In reality, communities put in place to drive local improvement maintained existing hierarchical structures and division of labour. Rules of engagement were defined by the top rather than negotiated and agreed by members. For example, Scottish and Welsh participants highlighted the imbalance in representation and control within perinatal optimisation and clinical groups which were led by neonatologists. Such power imbalances hindered maternity team staff motivation to engage as illustrated by the following excerpt:

*If I go to a (joint perinatal improvement) meeting where there is only two obstetricians and 12 neonatal consultants and 12 advanced neonatal practitioners and they are talking in terms which I am not familiar with, certain things I’ve never heard of, next time, when I have to prioritise (which meetings I can attend), I will say, I am probably not needed there. (P07, Obstetrician, Wales)*

Another challenge was relying on implementers’ good will to engage in activities without meaningful support from managers and organisations. Welsh and Scottish participants described participation in perinatal networks and collaboratives as sporadic and fragmented. They contrasted the experiences of devolved nation implementers with those of PReCePT and PERIPrem teams where champions were supported by backfill funding as reflected in the following excerpt:

*The difference with PERIPrem is that we were doing all this unfunded through the network, so people were doing it as additional roles, and the main challenge that we have had is […] It’s almost been different people on different meetings, and that’s been one of the biggest challenges. (P03, Neonatologist, Wales)*

Networks of participation and CoP which differed in form and function existed at different levels of the system: multi-professional clinical teams (adopters), perinatal implementer teams, regional and national perinatal clinical and improvement networks. Team capital and boundary spanners i.e. individuals linking these communities together, were important for scaling and spreading improvement. Participation in multiple networks or communities allowed access to different actors – e.g. patient and public representatives not routinely embedded in local QI activities – who could contribute to implementation activities, for example, by giving feedback to presentations. In the devolved nations shared governance and participation in networks and communities relied on individuals and teams, but PReCePT intentionally and strategically redistributed roles and responsibilities away from obstetricians and neonatologists to midwives, nurses and junior clinical staff, and placed midwives at the centre of implementation. Midwives, because of their “shop floor” presence at the intersect of maternity and neonatology were ideal boundary spanners and facilitators of knowledge co-creation. Working as part of a CoP was experienced as empowering by champions who felt appreciated and supported by their teams and organisation. Individual champion characteristics e.g. protected time, level of seniority and improvement competencies, and support from peers and managers, contributed to how successful they were however. Input and sanctioning from obstetric and neonatal leads was required for embeddedness in routine activities, and this required strategic planning to allow time and space for all three to come together and discuss the intervention, in this case within PReCePT CoPs as the following excerpt illustrates:

*We (the midwife* *champion, the obstetric and neonatal leads) started working together on the project from that day one learning event […]it almost made it easier to divide job roles* in *terms of responsibilities (P13, Midwife, Enhanced Support)*

### Collective action

Collective action refers to the operational work people do to support a new practice [17]. In this case, it refers to what members of the perinatal team do individually and collectively to ensure MgSO_4_ is administered, and data are captured accurately. It involves appropriate task allocation, workflow articulation, and collaboration among perinatal team members.

Administering MgSO_4_ involves multiple linked tasks: identifying women at risk of preterm labour, organising admission to labour ward, and sometimes transferring to a different setting according to “birth in the right place” pathways. While maternity teams were administering MgSO_4_ to some women, uptake varied significantly between and within units. Smaller units, not equipped for preterm care and lacking neonatal resource – impacted by funding and commissioning decisions—were found to be at a disadvantage and struggled to improve because of gaps in staff’s competencies and because they faced additional tasks on top of caring for women, such as organising in-utero transfers. Maternity teams had to make quick decisions and coordinate with neonatal teams for intervention eligibility and timings. Unless maternity and neonatal teams were working together from the moment women got in touch with maternity services, delays in communication and decision-making could hinder MgSO_4_ administration, as illustrated by the following excerpt:

*There sometimes is a difficult conversation with the obstetricians where they have got a lady on the antenatal ward. Say she’s 24 weeks. And they say, ‘[…] She’s not in labour.’ […] that lady doesn’t get steroids (or) magnesium sulphate and nor does she get transferred to a unit who could cope more effectively if the baby was delivered. And then what happens is […] this lady is now in established labour […] but it might be a little bit too late (to administer these interventions). (P06, ANNP, Wales)*

Implementation strategies had to augment a complex array of competencies across diverse clinical teams, specialties and settings, but some teams were better than others in implementing improvement. Participation of local implementers in communities within and across organisations enabled for rapid diffusion of innovation, such as PReCePT boxes, and access to products such as performance monitoring tools, proformas, and training modules, not otherwise available to teams in organisations lacking in capacity and resources. It also connected champions and their teams to knowledge and peer support held collectively by community members which they could access on physical or digital realms: e.g. through WhatsApp groups and other social media closed communities. Access to and adoption of these products expedited individual and collective behaviour change as illustrated by the following excerpts:

*What (the PReCePT) national team did was that they had a great basis and a communication system, particularly a simple thing as the toolkit being available and freely accessible on the internet with dedicated webpage. I know it’s sounds very straightforward and sounds very simple, but it made a huge difference’ (Regional Clinical Lead P05, NPP)*

### Reflexive monitoring

Reflexive monitoring is the appraisal work people do to assess and understand the ways a new set of practices affect them and others around them, and how well they think they are working to inform future improvement activities [17]. In this case, appraisal work refers to outcome measurement and audit activities taking place to assess adherence to MgSO_4_ clinical guidance and how this knowledge is fed back into the system.

Neonatal teams, as the statutory guardians of the clinical intervention, are required to capture MgSO_4_ uptake data in Neonatal Badgernet which informs the National Neonatal Audit Programme's (NNAP) annual reports. NNAP galvanised strategic and clinical leadership towards improvement activities but this did not always translate to mobilising staff on the ground. Analysis highlighted how performance knowledge was boundaried, as audit was carried out by neonatal teams and discussed by clinical leadership, but not routinely cascaded to staff on the ground. Sharing performance data with maternity teams on a yearly basis had a short term impact on improvement, and was not enough to sustain the change beyond time of feedback, as illustrated in the following excerpt:

*When something goes wrong people will concentrate on that. So, it will improve and after that they even take it for granted. […], then it (practice) goes back to the default. (P04, Neonatologist, Wales)*

Analysis highlighted the value of embedding performance discussions in formal and informal activities such as bedside and formal teaching activities, without the focus being on assigning individual responsibility. Sustained improvement required targeted efforts for continuous audit beyond that demanded by NNAP and relied on quality data and audit. Strategies such as missed case analyses, process mapping, and PDSA cycles, helped teams identify blockers, barriers and challenges and inform improvement plans. For example, missed case analyses highlighted poor quality of data inputted in Budgernet. Data quality and measurement were a challenge across the board: in Scotland, a separate data collection system for MCQIC was in place alongside Badgernet and staff needed to input the same data in both. This added to staff workload and hindered audit and assessment. Simplifying data collection systems was therefore a strategic priority in Scotland as illustrated in the following excerpt.

*Something that we’re working on within the new programme is to get rid of this duplication of measurement, and actually pull it off from the one source that it’s all going into, and that’s definitely a priority that we’ll hopefully take forward with the new part of the programme. (P13, Neonatologist, Scotland)*

Being able to communicate weekly performance to staff was part of the strategies used to embed the intervention in practice, and ensure it was on staff’s mind, but there were structural and cultural barriers to that e.g. lack of shared governance, digital system limitations, and no requirement for data to be shared beyond the yearly NNAP report. Participants described the use of tools and strategies aligning with PReCePT methodology such as digital tools to transform weekly data into posters, as the following excerpt illustrates:

*I still feel as if there’s a lack of sharing data, in a bit more of a meaningful way to front-line teams […] what we started to do locally is generate a […] poster. […] it’s found to be a bit more meaningful to teams. They definitely respond a little bit more to something a bit more visual than a run chart. (P13, Neonatologist, Scotland)*

For system wide change to happen and be sustained, local audit was not enough. Connections between actors and teams across the perinatal ecosystem, for example through learning communities overseen and supported by improvement leads (alongside backfill funding to protect time) were also essential and helped address geographical and organisational disparities in improvement capability. Managerial oversight and facilitation of such communities, as provided by the Enhanced Support intervention of the PReCePT study helped with strategic planning for improvement, and capacity-building in the system, and this approach informed the design of PERIPRem as illustrated in the following excerpt:

*(PReCePT champions) had the PReCePT toolkits, but none of that was QI training. […] For PERIPrem, we had lined up that […] everyone was going to be upskilled to silver level ambassador training, because we just felt that last time […] It was a very light touch, and it required an awful lot of self-educational want from the person who had taken up (the PReCePT champion role). (AHSN manager P11, NPP)*

## Discussion

### Overview of findings

We used findings from three studies investigating implementation of MgSO_4_ guidance in England, Scotland and Wales. National and professional perinatal networks and collaborative communities of practice set up by these networks were instrumental for sharing knowledge across systems and organisations, and resulted in toolkits, products and approaches diffusing across nations. Implementation efforts focused on communication channels between maternity and neonatal teams and creating a shared sense of understanding to help mobilise members of the perinatal team in planning for and administering the intervention and improving data collection for continuous improvement.

What was different between the three nations at the time of data collection was the role of funded midwifery champions acting as boundary spanners and linking actors across levels of the eco-system. Another difference were the regional, intervention-specific CoPs put in place by the National PReCePT Programme and Study as a platform for implementation champions to share knowledge, peer support, and co-deliver the intervention. Even though in Scotland a centrally led effort to implement MCQIC created collaboration and learning opportunities during early stages of implementation, responsibility was subsequently passed onto regional health boards which removed the links with and oversight by QI and clinical leadership.

On top of that, enrolment of obstetric and neonatal champions with protected time alongside funded midwife champions, and opportunities for implementation capacity-building in midwives drove system-wide change by creating KMb resource within settings and organisations, for example multi-professional communities of practice, changes in policies, documentation, leadership models, clinical and audit systems, and how perinatal clinical microsystems are organised and operate, e.g. shared governance. We use these findings to provide recommendations on successful strategies used to maximise success of scaling programmes and interventions (Table 2).

**Table 2:** Recommendations for scaling and spreading evidence-based interventions

**1. Assess Implementation Readiness** • Conduct a thorough assessment of each setting’s resources, including staffing, data infrastructure, and cultural readiness • Identify settings with low socio-cognitive and socio-structural resources and prioritize them for additional support
**2. Provide Targeted Support** • Offer additional coaching, mentoring, and resources to settings identified as needing more support to align their performance with better-performing units • Ensure that all teams have access to quality improvement (QI) training and resources, especially those in resource-strained settings
**3. Strengthen Collaborative Communities of Practice** • Foster the development of interdisciplinary collaborative communities of practice that include maternity, neonatal, public involvement, safety and improvement stakeholders • Promote distributed leadership within these communities to ensure equal participation and knowledge-sharing across all roles and disciplines • Embed capacity building opportunities within collaborative communities
**4. Enhance Data Infrastructure** • Improve data infrastructure to ensure that data is accurate, timely, and meaningful for all actors involved in implementation • Facilitate effective communication of data across all levels of the system to inform ongoing improvement efforts
**5. Ensure Protected Time for Champions** • Recognize the importance of champions as crucial facilitators and brokers of knowledge and support their active engagement • Secure funding and organizational support to provide champions with protected, backfilled time for participating in implementation activities
**6. Promote Equity in Implementation** • Implement strategies to ensure equitable distribution of resources and opportunities across all settings, especially those facing greater challenges • Understand issues of power and agency among actors which impact on ability to take advantage of opportunities and mobilise resources • Embed participatory approaches and co-production methods to ground implementation efforts within an equity and justice framework
**7. Monitor and Evaluate Progress** • Continuously monitor the impact of interventions and the effectiveness of collaborative communities • Conduct longitudinal evaluations to measure not only clinical outcomes but also the sustainability of interventions and the adaptability to new knowledge
**8. Scale and Spread Successful Practices** • Identify and document successful practices and innovations to facilitate their scaling and spread across different settings • Ensure that any scaling efforts are accompanied by opportunity- and capability- boosting interventions for teams most in need
**9. Foster Strong Perinatal Leadership** • Develop strong perinatal leadership to drive the creation of alliances, ensure equitable power distribution, and cultivate a shared sense of culture and commitment within the collaborative communities
**10. Plan for Continuous Improvement** • Embed continuous improvement strategies within the system to sustain and refine interventions over time • Encourage reflexive monitoring and feedback loops to adapt interventions as new challenges and opportunities arise

### Situating findings in the literature

MgSO_4_ uptake in Scotland and Wales improved over time, yet concerns about performance disparities between settings drove improvement efforts. Addressing performance gaps between and within settings, particularly when it comes to vulnerable populations, is crucial for addressing health inequities but these are often hidden within implementation science research [30]. Co-designed QI Toolkits shared within CoP and networks expedited diffusion of innovation and reduced implementation capacity disparities between settings [31, 32]. However, without diverting resources to and creating capacity in local teams that needed it, teams relied on local contexts which effected implementation in different ways [3, 33–35] thus exacerbating health and performance inequities [36, 37]. Settings where staff demonstrate more knowledge, positive attitudes, and competencies (i.e. social-cognitive resources), and function within organisations rich in policies, teamworking and improvement culture, and high in material and workforce assets (social- structural resources) tend to demonstrate safer maternity practices [38]. This highlights a cycle of inequity whereby settings with the greatest need for improvement may lack the resources to engage in improvement activities.

Social structures – clinical networks, collaboratives and CoP – provided a platform for knowledge and innovation to be shared within the system and reach teams in resource-poor organisations. Such groups acted as improvement scaling and spreading mechanisms through opportunities to share innovation, constantly re-negotiate the clinical intervention, how it should be operationalised in practice, and collaborating in its implementation. Knowledge exchange between experts and knowledge users can allow responsiveness to context and knowledge adaptation to address needs of implementers and adopters in frontline care, a necessary step for bridging the gap between implementation research and implementation practice [6, 39]. Midwifery champions were ideally placed to adapt implementation according to their contexts as conduits of knowledge between their teams and perinatal optimisation interest holders across the system, but needed the right personal attributes and organisational support to do so [40].

Existing relational infrastructure e.g. perinatal clinical and safety and improvement networks, collaboratives and communities, facilitated the implementation of MgSO_4_ by allowing local implementers to access expertise and training specific to the intervention, but not all were able to access or utilise these opportunities. A key PReCePT finding was the role of intervention-specific CoP facilitated by senior leadership, and with input from clinical leads and QI experts [20, 41]. Such CoP creating improvement capacity, nurtured perinatal partnerships and co-creation of knowledge but for higher order KMb capabilities [18] to be achieved capacity-building in members is essential. Team capital [42] – teams that can draw from the skills, resources, networks and alliances of members from a diverse range of disciplines and clinical and non-clinical roles – is thus placed at the centre of CoP for KMb capacity-building.

Findings reiterate the value of good quality performance data to inform continuous assessment and improvement. Robust data infrastructure, and cross-system communication and learning are central to Learning Health Systems [43]. Learning Health Systems (LHS) have been defined as “a team, provider, or group of providers that, working with a community of stakeholders, has developed the ability to learn from the routine care it delivers and improve as a result” [43]. LHSs align with the function of CoPs: a community with a shared focus of interest continually renegotiated by members, mutual engagement, and shared repertoire of communal resources [44]. However, findings also highlight how a focus on data collection without ensuring this is relevant, meaningful, and fits in existing workflows can hinder improvement.

## Strengths and limitations

We interviewed PReCePT implementers (champions, clinical leads, and QI managers) in England, and strategic and clinical leads and implementers in Scotland and Wales to understand implementation experiences. Strengths and limitations of the NPP and cRCT Study qualitative evaluations have been reported [11, 13, 16]. In the devolved nation study we only interviewed strategic and clinical leads in Scotland and Wales whose perspectives may not fully represent frontline clinical care staff. Additionally, participants volunteering to participate in the study may have been experienced in QI, and/or working in settings high in improvement capability compared to those not coming forward. We also acknowledge the voice of service users is absent from this study.

Analysis highlights how KMb helped set in motion NPT implementation mechanisms which operated in an iterative, dynamic, and interconnected way. This research contributes to the NPT literature by illustrating how implementation work must engage actors from all levels of the perinatal ecosystem, and relies on knowledge-sharing and collaboration within diverse networks and CoPs [45]. Current debates within the scaling and spreading of improvement literature discuss the need for intervention fidelity versus adaptability [46]. Findings suggest the usefulness of NPT as a theory of change denoting the *function* of improvement interventions as opposed to process fidelity. In this sense, our work situates the NPT as a useful framework for aligning implementation science with improvement practice, allowing for tailoring of implementation interventions to fit the culture, infrastructure, and practice of specific health care systems. Analysis also adds to the theory by demonstrating the impact on staff wellbeing of implementation strategies to address power imbalances and thereby enhance staff agency and empowerment e.g. capacity-building, distributed leadership and governance.

Further research is needed to understand how KMb communities can not only facilitate implementation, but also support sustainment, discontinuation or tailoring of interventions to accommodate for new evidence being produced. This would require longitudinal process evaluation studies, and measuring outcomes beyond clinical performance.

## Conclusions

Policy and evidence for clinical interventions can galvanise support and backing for implementation interventions. Yet they do not always mobilise actors into action equitably across settings, or result in sustained improvement because of contextual differences in implementers’ ability to access and mobilise social-cognitive and social-structural resources for improvement. QI interventions driven by KMb can drive scaling and spreading of improvement, but require knowledge sharing and infrastructure within the system to support improvement capacity building, as well as strong leadership and addressing power imbalances between co-actors. Protected funding is also necessary for local champions to create capability and capacity to become boundary spanners, linking communities across the perinatal optimisation eco-system and lead on local improvement.

## Supplementary Information

Supplementary Material 1.

## Data Availability

The datasets generated and/or analysed during the current study are not publicly available to protect the anonymity of staff and their employers but are available from the corresponding author on reasonable request.

## Abbreviations

AHSNs: Academic Health Science Networks
ANNP: Advanced Neonatal Nurse Practitioner
BAPM: British Association of Perinatal Medicine
CoP: Communities of Practice
cRCT: Cluster Randomised Controlled Trial
KMb: Knowledge Mobilisation
LHS: Learning Health Systems
MatNeo SIP: Maternity and Neonatal Safety Improvement Programme
MCQIC: Maternity and Children Quality Improvement Collaborative
MgSO_4_: Magnesium Sulphate
NHS: National Health Service
NICE: National Institute for Health and Care Excellence
NNAP: National Neonatal Audit Programme
NPP: National PReCePT Programme
NPT: Normalisation Process Theory
PERIPrem: Perinatal Excellence to Reduce Injury in Premature Birth
PPWP: Preterm Perinatal Wellbeing Package
PReCePT: Prevention of Cerebral Palsy in PreTerm Labour
QI: Quality Improvement
SPSP Perinatal: Scottish Patient Safety Programme Perinatal
UK: United Kingdom
