# Supplementary material for: Lessons from using the Normalisation Process Theory to understand adherence to guidance on MgSO4 in preterm labour
Source: Implement Sci Commun. 2025 Jul 16;6:75. doi: 10.1186/s43058-025-00758-1 (PMC12265282; doi:10.1186/s43058-025-00758-1)
Supplement: Supplementary file 1 — Supplementary Material 1. [file 43058_2025_758_MOESM1_ESM.docx]

| Nation |  | National interventions/strategies | Intervention aim | Intervention components | Implementation leadership |
| --- | --- | --- | --- | --- | --- |
| England | 2014-2015 | PReCePT codesigned and piloted in the West of England | - Use QI methodologies to codesign (clinicians, parents, QI experts) and implement and QI intervention |  | AHSN West of England  5 NHS Trusts in West of England |
|  | 2018 | National PReCePT Programme | - Scale up the PReCePT intervention | - PReCePT QI Toolkit - PReCePT implementation guidance - QI training sessions - Regional support - Local obstetrician champion - Funded local midwife champion | AHSNs  PReCePT NPP steering group |
|  | 2018 | PReCePT study | - Compare the effectiveness of a Standard versus Enhanced support model of implementation | - Standard Support arm as above - Enhanced Support arm as above PLUS - PReCePT “how to” guides - Funded local neonatal champion - Structured coaching from QI coach; personalised, and as part of a regional QI collaborative - Funding for up to three local champions to attend learning events | PReCePT Study steering group |
|  | 2020 | PERIPrem (Perinatal Excellence to Reduce Injury in Premature Birth) | Uses PReCePT methodologies for the implementation of a perinatal care bundle of 11 interventions (including MgSO4) that demonstrate a significant impact on brain injury and mortality rates amongst babies born prematurely | - Downloadable Implementation Resources - Downloadable PERIPrem baby and clinical passports | Health Innovation West of England, Health Innovation South West  Resources available from HIWoE and BAPM websites |
| Scotland | 2017 | Preterm Perinatal Wellbeing Package (PPWP) | Improve perinatal quality and safety of care through a bundle of interventions during preterm labour; includes posters and guides; MgSO4 is one of the core measurement indicators | - Initially nationally led by MCQIC team with involvement from QI leads - Regionally there was funding for MCQIC midwives but this was phased out - Support included visits from MCQIC coaches to clinical leads in regional health boards - webinars - national meetings - A Core Measurement Plan and data collection system - expectation of performance reports prepared by neonatal teams which were then fed back by coaches - Clinical teams could choose which indicators to focus on. - Implementation responsibility was eventually placed on Health Boards | Maternity and Children Quality Improvement Collaborative (MCQIC), Scottish Patient Safety Programme (SPSP)  Now renamed into Scottish Patient Safety Programme’s (SPSP) Perinatal Programme |
| Wales | At time of data collection | - Embed national and professional guidance in Welsh clinical guidance and pathways - Create cross-organisational horizontal partnerships and networks - Disseminate knowledge to local units to drive collective action |  | - Perinatal Optimisation Task and Finish Group - Annual network audit and QI meetings for network members - A strategic push for perinatal teamworking - Use of existing Toolkits, products and innovation e.g. PReCePT/PERIPrem, BAPM perinatal optimisation; perinatal teamworking toolkits |  |
|  | 2023 | PERIPrem Cymru | Uses PReCePT methodology to improve uptake of 11 evidence-based interventions in preterm birth. | - Downloadable PERIPrem implementation resources - PERIPRem champions | Welsh Joint Maternity and Neonatal Strategic network  Welsh Government |
| National | 2020 | Antenatal Optimisation for Preterm Infants less than 34 weeks A Quality Improvement Toolkit | Provides tools and resources to support QI leads in their perinatal optimisation efforts. Includes MgSO4. | - Provides a pathway to improvement using PDSA principles - Provides documentation to use as part of training, raising awareness, proformas, etc. | British Association of Perinatal Medicine |
